# Editorial: Improving diagnosis and management of genetic lipodystrophy

**DOI:** 10.3389/fendo.2024.1543126

**Published:** 2025-01-23

**Authors:** Milena Teles, Rinki Murphy

**Affiliations:** ^1^ Department of Endocrinology and Metabolism, Clinical Hospital, Faculty of Medicine, University of São Paulo, São Paulo, Brazil; ^2^ Department of Medicine, Federal University of Ceara, Fortaleza, Brazil; ^3^ Núcleo de Pesquisa e Desenvolvimento de Medicamentos (NPDM), Department of Physiology and Pharmacology, Federal University of Ceará, Fortaleza, Ce, Brazil; ^4^ Faculty of Medical and Health Sciences, The University of Auckland, Auckland, New Zealand; ^5^ Specialist Weight Management Service, Te Mana Ki Tua, Counties Health New Zealand, Te Whatu Ora, Auckland, New Zealand

**Keywords:** lipodystrophy, diagnosis, genetics, heterogeneity, LMNA, PPARG, congenital generalized lipodystrophy (CGL), partial lipodystrophies

This Research Topic explores the theme *“Improving Diagnosis and Management of Genetic Lipodystrophy”*, presenting five innovative articles describing hereditary lipodystrophies. These careful observations characterize the phenotypic heterogeneity across the lifespan in gestational, neonatal outcomes, and later life morbidity and mortality. All document the female predominance in identification, with males largely diagnosed through cascade testing. These studies are particularly relevant for enhancing the early detection, cascade screening and management of this rare genetic condition.

The article by Guidorizzi et al., *Comprehensive analysis of morbidity and mortality patterns in familial partial lipodystrophy patients*, examines patterns of morbidity and mortality in 106 patients (78% female) with familial partial lipodystrophy (FPLD) in Brazil with genetic confirmation. The study highlights the prevalence of genetic variants, such as LMNA (85.8%) and PPARG (10.4%), as major etiological factors. Metabolic comorbidities were predominant, including diabetes mellitus (57.5%) and metabolic-associated fatty liver disease (56.6%) in the presence of partial subcutaneous body fat loss as determined by low caliper skinfold thickness on the anterior thigh (<10mm for men and <22mm for women). Based on a large cohort of patients, this study offers a detailed characterization of FPLD and lays the foundation for improved clinical management.

Soares et al., in *Familial partial lipodystrophy resulting from loss-of-function PPARγ pathogenic variants*, investigate the phenotypic and clinical characteristics in carriers of 41 different loss-of-function variants in the PPARγ gene (FPLD3) affecting 91 patients (76% female). The study underscores clinical heterogeneity, with high prevalence rates of metabolic comorbidities such as diabetes (77%), hypertension (59.5%), and metabolic-associated fatty liver disease (87.5%), again in the presence of partial fat loss in the gluteal and/or lower limbs. These findings reinforce the significance of the PPARγ gene in adipose tissue metabolism and the need to deepen genetic understanding of this condition.

Valerio et al., in their study *Gestational and neonatal outcomes of women with partial Dunnigan lipodystrophy*, explore obstetric complications in 17 pregnancies occurring in 8 women with FPLD2 due to *LMNA* variants. Key findings include gestational diabetes in 25% of patients and preeclampsia in 12.5%. Additionally, neonates exhibited high rates of macrosomia (29.4%) and hypoglycemia. This work highlights the importance of careful pregnancy planning and management to minimize complications in women with lipodystrophy.

The study by Fernández-Pombo et al., *Natural history and comorbidities of generalized and partial lipodystrophy syndromes in Spain*, examines the natural history of generalized (n=24) and partial lipodystrophies (n=91), with 58% women in the former but 82% in the latter, suggesting that generalized cases are easier to identify in both men and women, than partial cases of fat loss which are more apparent in women. The mean delay in diagnosis was 7 years for generalized and 24 years for partial lipodystrophies from symptom onset. This longitudinal study provides valuable data on the prevalence and progression of these conditions in a diverse population, contributing to the global understanding of these diseases.

Finally, Freire et al., in *Heterogeneity and high prevalence of bone manifestations, and bone mineral density in congenital generalized lipodystrophy subtypes 1 and 2*, investigate bone characteristics in patients with congenital generalized lipodystrophy (CGL) subtypes 1 and 2. Findings include common osteolytic lesions (74%) and osteosclerotic lesions (42%), as well as elevated bone mineral density (68.4%). The study emphasizes the need for systematic evaluation of bone manifestations in CGL patients and the importance of understanding the pathogenesis of these abnormalities.

Collectively, the articles in this Research Topic provide a comprehensive overview of hereditary lipodystrophies, addressing challenges such as phenotypic heterogeneity and genotype-phenotype correlations, along with strategies for clinical management.

